# Red-Light Transmittance Changes in Variegated *Pelargonium zonale*—Diurnal Variation in Chloroplast Movement and Photosystem II Efficiency

**DOI:** 10.3390/ijms241814265

**Published:** 2023-09-19

**Authors:** Sonja Veljović Jovanović, Bećko Kasalica, Katarina Miletić, Marija Vidović, Nikola Šušić, Dejan Jeremić, Ivan Belča

**Affiliations:** 1 Institute for Multidisciplinary Research, University of Belgrade, 11030 Belgrade, Serbia; nikola.susic@imsi.bg.ac.rs; 2 Faculty of Physics, University of Belgrade, 11001 Belgrade, Serbia; kasalica@ff.bg.ac.rs (B.K.); katarinamiletic@ff.bg.ac.rs (K.M.); bivan@ff.bg.ac.rs (I.B.); 3 Institute of Molecular Genetics and Genetic Engineering, University of Belgrade, 11042 Belgrade, Serbia; mvidovic@imgge.bg.ac.rs; 4 Innovation Center of the Faculty of Chemistry, University of Belgrade, 11001 Belgrade, Serbia; djeremic@chem.bg.ac.rs

**Keywords:** chloroplast movement, red-light transmittance, dark–light transition, blue-light receptors, phototropins, variegated *Pelargonium zonale*

## Abstract

Chloroplast movement rapidly ameliorates the effects of suboptimal light intensity by accumulating along the periclinal cell walls, as well as the effects of excess light by shifting to the anticlinal cell walls. These acclimation responses are triggered by phototropins located at the plasma membrane and chloroplast envelope. Here, we used a recently developed non-invasive system sensitive to very small changes in red light leaf transmittance to perform long-term continuous measurements of dark–light transitions. As a model system, we used variegated *Pelargonium zonale* leaves containing green sectors (GS) with fully developed chloroplasts and achlorophyllous, white sectors (WS) with undifferentiated plastids, and higher phototropin expression levels. We observed biphasic changes in the red-light transmittance and oscillations triggered by medium intensities of white light, described by a transient peak preceded by a constant decrease in transmittance level. A slight change in red-light transmittance was recorded even in WS. Furthermore, the chloroplast position at lower light intensities affected the rapid light curves, while high light intensity decreased saturated electron transport, maximum quantum efficiency of photosystem II, and increased non-photochemical quenching of chlorophyll fluorescence and epidermal flavonoids. Our results extend the knowledge of light-dependent chloroplast movements and thus contribute to a better understanding of their role in regulating photosynthesis under fluctuating light conditions.

## 1. Introduction

Most of the incident radiation in the visible part of the electromagnetic spectrum reaching the leaf surface is absorbed by the photosynthetic pigments (chlorophyll, carotenes and xanthophylls). The chlorophyll (chl) absorbs most of the blue and red light and is responsible for the low reflectance in the visible spectral range and the green colour of the leaf. In addition to chl fluorescence, all other leaf optical properties, such as transmittance, absorbance and reflectance, are extensively studied in plant physiology (photosynthesis, photomorphogenesis), plant-environment interactions and remote sensing [1,2,3,4]. Under ambient conditions, plants are often exposed to sudden fluctuations in light intensity. This has led to the development of numerous mechanisms aimed at either (i) avoiding photoinhibitory conditions induced by excess light (energy that cannot be completely used in photosynthesis), or (ii) enhancing a photosynthetic photon efficacy under suboptimal light intensities.

Excess light has a damaging effect on the photosynthetic apparatus and plants as a whole [5,6]. One of the most rapid protective mechanisms against excess light is the development of the proton gradient across the thylakoid membrane [7,8] and violaxanthin deepoxidation to zeaxanthin [9]. Both the trans-thylakoid proton gradient and zeaxanthin formation lead to dissipation of excess excitation energy in the photosystem II (PSII) by heat, resulting in non-photochemical quenching (NPQ) of chl a fluorescence [10]. The additional means of avoiding photodamage is the short-term and reversible redistribution of absorbed light energy between the two photosystems, a process called state transitions, which is associated with changes in the phosphorylation of the light-harvesting complex that determine binding to PSII or PSI [11,12]. The alternative pathways of photosynthesis, such as the Mehler reaction, nitrogen and sulphur assimilation, and the malate valve, [13,14] may contribute to the dissipation of the excess of absorbed photons depending on physiological and environmental conditions. Recently, it has been proposed that the alternative electron sink—the phenylpropanoid pathway—may also be regulated by a specific protein, TROL, which is either closely associated with PSI or released [15].

Besides the above mechanisms regulating light capture, plants developed a highly controlled mechanism—the relocation of chloroplasts within the cell in response to the intensity and direction of blue light [16]. While in angiosperms chloroplast movement is regulated by blue-light receptors, in ferns it is regulated by red light [17]. Moreover, it has been demonstrated that in Arabidopsis both processes—chloroplast movement and phototropism—were triggered by red-light irradiation through phototropins, [18,19]. The chloroplasts shift towards the irradiated areas within the cell (periclinal to the direction of actinic light) to efficiently absorb the light and increase photochemical efficiency (the accumulation response). On the other hand, as part of a photoprotective mechanism, chloroplasts move away from excess light towards the anticlinal cell walls to avoid photo damage (the avoidance response) [20,21,22,23]. In addition to the photoprotective role, Wilson and Ruban [24] proposed the alternative role of the avoidance response, which could enhance light penetration into deeper mesophyll layers. Although the phenomenon of light-induced chloroplast movement was discovered more than a century ago [25,26,27], reviewed by [28], the involvement of the specific photoreceptors, phototropins (Phot1 and Phot2), in this process was discovered two decades ago [29,30]. The phototropin 1 encoding gene *phot1*, which has been shown to control phototropism, was first isolated in Arabidopsis [31]. In addition to phototropism and chloroplast movement, Phot1 and Phot2 have been shown to play a role in controlling the stomatal opening in Arabidopsis [32]. Thus, the phototropin family of photoreceptors plays an important role in photomorphogenesis, being responsible not only for phototropism, stomatal opening and chloroplast relocation, but also for leaf flattening [33,34,35]. Its role as a mediator of the chloroplast accumulation response was evidenced in a study with the *phot1phot2* Arabidopsis mutant, since both Phot1 and Phot2 redundantly control chloroplast avoidance [36].

It has been shown that Phot2 was responsible for chloroplast avoidance under strong light and blue-light intensities >20 μmol photons m^−2^ s^−1^ through autophosphorylation, which is the primary step of all phototropins. Phot1 and Phot2 are located in the plasma membrane and Phot2 in the chloroplast envelope, and although both are triggered by low blue light (0.01 to 20 μmol photons m^−2^ s^−1^), they exhibit different efficiencies. Phot1 is more sensitive to lower light intensity, while Phot2 is only activated from 2 μmol photons m^−2^ s^−1^ [29,30]. 

Although the specific role of Phot1 and Phot2 in chloroplast translocation is well documented, and the associated molecular mechanism of this process has been thoroughly characterised, there are still puzzling parts of the underlying downstream signalling of chloroplast movement. As for the physiological significance of this process, one of the questions is attributed to the relationship between cell position of chloroplasts and the regulation of photosynthesis, as a part of the acclimatisation to naturally occurring sun exposure fluctuations. There is a growing interest in studying the effect of chloroplast position on light absorption and excitation pressure in PSII, as well as on the prevention of photoinhibition damage under excess light [24,37].

Wada and Kong [38] have described several methods for measuring and analysing the accumulation and/or avoidance response, ranging from naked-eye detection of pale or dark green leaf sections, to a conventional microscopic evaluation of fixed leaf sections, to an optical method for measuring red-light transmittance. Each method has its advantages and limitations, but the most widely used method for studying chloroplast movement is the measurement of leaf optical changes by absorption spectrophotometry, which was first used by Inoue and Shibata [39]. In addition, chloroplast movement in Arabidopsis transgenic lines was studied using fluorescent markers with confocal laser scanning microscopy and/or fluorescence microscopy with total internal reflection [19]. Recently, we described a non-invasive measurement system to continuously monitor and record the optical transmission of leaves at minute time intervals throughout the day and even over weeks [40]. The method described is applicable to various plant species, with a leaf holder allowing five degrees of freedom for mechanical movement under different leaf positions and orientations. The method provides information on very small changes in chloroplast movement, indistinguishable by most other methods [38].

To test the hypothesis that chloroplast movement plays an essential role in regulating photosynthetic electron transport, we simultaneously measured the dynamics of PSII photochemistry and NPQ and compared them with transmittance changes during dark–light–dark transitions in the leaves of variegated *Pelargonium zonale*. These leaves contain white leaf sectors (WS) at the leaf margin and photosynthetically active green leaf sectors (GS) in the leaf centre. Our aim was to investigate: (1) the contribution of chloroplast movement to photochemical efficiency of PSII, (2) the transmittance of red light through WS of *P. zonale*, and (3) the complex, oscillatory and rapid transmittance changes triggered by different light intensities possibly indicating yet unknown mechanism of regulation between two opposing chloroplast responses triggered by sudden illumination. 

## 2. Results

### 2.1. Changes in the Red-Light Transmittance and chl Fluorescence Changes at Dark–Light–Dark Transitions in Green Leaf Sectors

**Figure 1:**
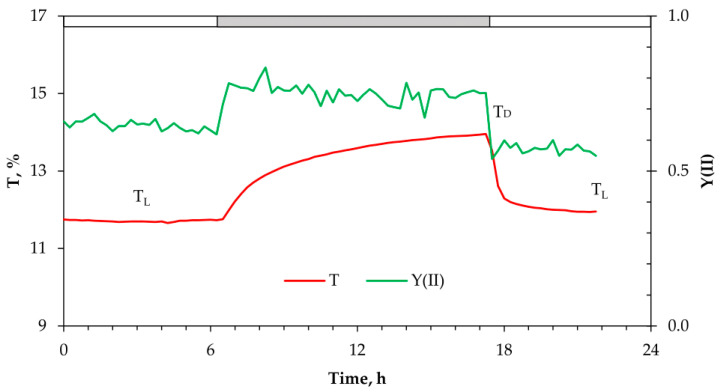
White-light-induced changes in red-light transmittance (665 nm) expressed as a percentage of total T and Y(II) (calculated as (F_M_′ − F)/F_M_′), determined at the same life spot during light–dark–light transitions according to the circadian rhythm. The intensity of white light was ~150 µmol photons m^−2^ s^−1^ at the leaf level. The proportion of blue light was 13%. T_D_—red-light transmittance in the dark before switching on the actinic light; T_L_—red-light transmittance at the end of the light period; ΔT_DL_—difference in red-light transmittance between dark and light levels. The diurnal T changes under light intensities of 25–150 μmol photons m^−2^ s^−1^ were reversible and repeatable over several days and weeks (Supplementary Figures S1 and S2).

The aim of our study was to investigate a possible relationship between chlorophyll (chl) fluorescence parameters and chloroplast movement within leaf cells. When variegated *P. zonale* plants were illuminated with a lower white light intensity (140 μmol photons m^−2^ s^−1^), the red-light transmittance (T) of GS of the attached leaves started to decrease immediately from dark level (T_D_) and reached at least 80% of the final steady-state level (T_L_) within one hour (Figure 1 and Figure S1). The difference between these two levels (ΔT_DL_, Figure 1) may vary among plants, mainly due to their different developmental stage. The time course of the observed changes in the red-light transmittance during dark–light–dark transitions is characterised by two phases: the first, fast phase, lasting less than one hour, and the second, slow phase, where a plateau is reached after several hours in the light. The transmittance’s reverse to the final T_D_ took several hours in the dark. In parallel to the transmittance measurement, we continuously recorded the modulated chl fluorescence. We showed that the effective photochemical quantum yield of PSII attained its final values within 20 min (Figure 1 and Figure S2), regardless of whether it is light or dark. This is much faster than the T changes measured at the same leaf spot.

**Figure 2:**
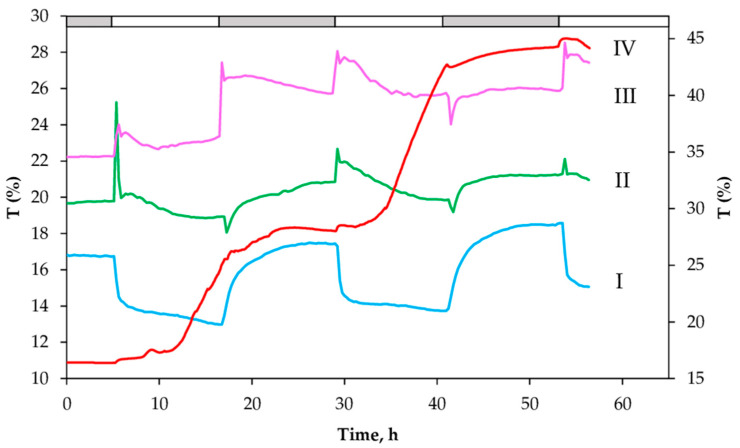
The representative curves showing diurnal changes in the red-light (665 nm) transmittance (T) expressed as a percentage of total transmittance through green sectors of leaves. Transmittance was recorded every 15 min. During the three-day measurement of T, *P. zonale* plants were grown at four light intensities: I: 25 μmol photons m^−2^ s^−1^; II: 290 μmol photons m^−2^ s^−1^; III: 350 μmol photons m^−2^ s^−1^; IV: 1200 µmol photons m^−2^ s^−1^ (shown on secondary axis). Dark periods (12 h) are shown in grey and light periods in white. Three replicates (from three different plants) are shown in Supplementary Figure S3.

However, unlike simple kinetic T changes induced at light intensities of less than 150 μmol photons m^−2^ s^−1^ (Figure 2 and Figure S3), more complex kinetics were obtained when the plants were grown at light intensities of >250 to 500 μmol photons m^−2^ s^−1^ (Figure 2). Thus, with increasing actinic light intensity, instead of a decrease in T, a transient increase in T was recorded as a peak, followed by a slow phase of T decreasing, similar to the trend observed at low light intensity. This change was similar in magnitude and kinetic properties (Figure 2) but opposite in direction to the change observed in plants at lower light intensity. At the highest light intensity (1200 ± 80 μmol photons m^−2^ s^−1^), T increased continuously from T_D_ to T_L_, without returning to the initial T_D_ value. Therefore, ΔT_DL_ is reversed compared to the changes at low light intensity (Figure 2 and Figure S3).

### 2.2. Changes in the Red-Light Transmittance and chl Fluorescence Changes at Dark–Light–Dark Transitions in White Leaf Sectors (WS)

**Figure 3:**
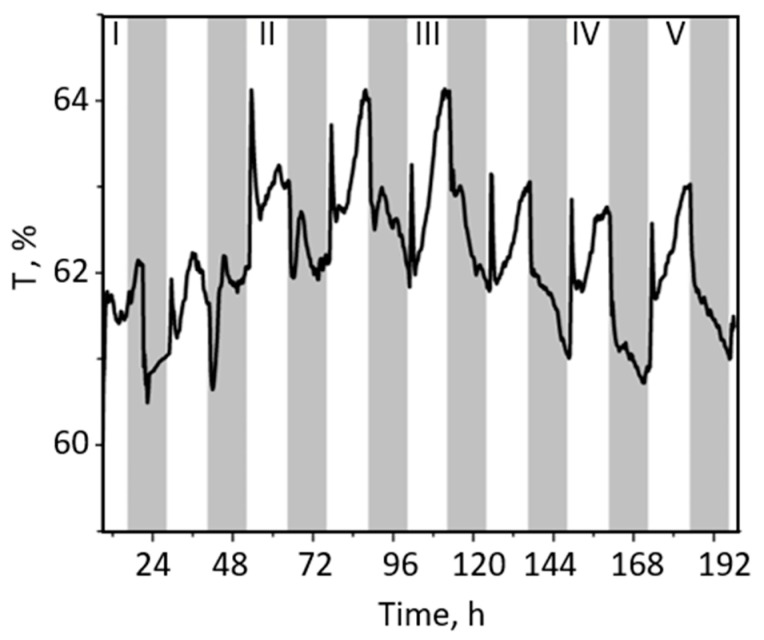
The representative curves showing diurnal changes in the red-light (665 nm) transmittance (T) expressed as a percentage of total transmittance of white sectors of leaves. Transmittance was recorded every 15 min. During the two-day measurement of T, *P. zonale* plants were grown at five light intensities: I: 25 μmol photons m^−2^ s^−1^; II: 140 μmol photons m^−2^ s^−1^; III: 290 μmol photons m^−2^ s^−1^; IV: 350 μmol photons m^−2^ s^−1^; V: 1200 µmol photons m^−2^ s^−1^. Dark periods (12 h) are shown in grey and light periods in white. The additional replicates (from three different plants) are shown in Supplementary Figure S4.

Figure 3 shows the changes in T induced by white actinic light of different light intensities in WS. Although the T level through white paper (as a control) was only twice as low compared to T of WS, the kinetics of the T changes in WS were repeatable, albeit with different kinetics than in GS. Interestingly, the negligible T changes in WS, detected during the dark–light transition, consisted of two peaks in the light, a fast and a slower increase (avoidance response), which were the opposite of GS. Upon darkening, T decreased regardless of the light regime (Figure 3 and Figure S4).

The obtained dynamics of T changes triggered by medium light intensities (ML, 250 to 500 μmol photons m^−2^ s^−1^) was more complex and involved two phases before the final T_L_ and T_D_ levels were reached, with smaller ΔT_DL_ (Figure 3 and Figure S4). After the first T peak, a small transient change in T was recorded, followed by the attainment of the steady state, implying an oscillatory regulation of T changes, with a period of about 40 min (estimated by the first derivative of the observed changes, Figure 2). In summary, ΔT_DL_ is a function of light intensity. Interestingly, in addition to the light-induced T peak increase, the transition from light to dark also triggered a transient, rapid T drop in plants previously exposed to a white light intensity of less than 500 µmol photons m^−2^ s^−1^. 

### 2.3. Chlorophyll Fluorescence Parameters

**Figure 4:**
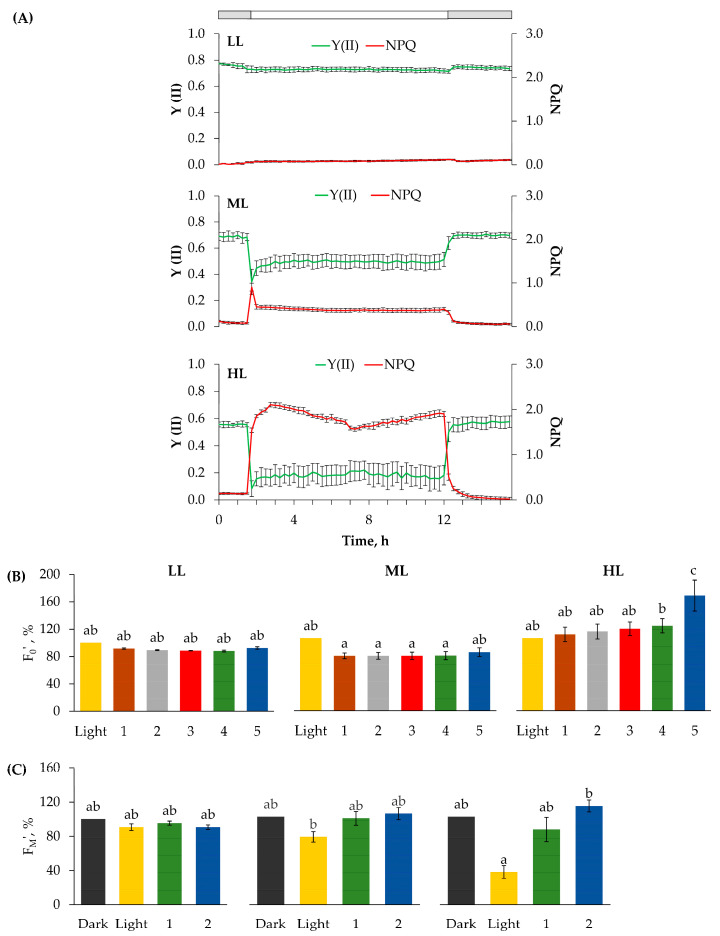
Chlorophyll fluorescence measurements. (**A**) Continuous measurements during 24 h of modulated chl fluorescence and derived photochemical efficiency Y (II) with NPQ at three light intensities: Low light, 100 µmol photons m^−2^ s^−1^; medium light, 350 µmol photons m^−2^ s^−1^; high light, 1400 µmol photons m^−2^ s^−1^. A mean of three curves, each consisting of measurements at 15 min intervals (±standard error), are presented. Dark periods are shown in grey, and light periods are shown in white. (**B**) F_0_′ were recorded at six time points during light–dark transitions: 0—immediately after the light was turned off; 1—two minutes after the light was turned off; 2—four minutes after the light was turned off; 3—seven minutes after the light was turned off; 4—ten minutes after the light was turned off; and 5—5 h after the 12-h light period. The mean values of points 1–5 were normalized and presented as percentages in relation to zero point (mean values of three measurements ± SE) (**C**) F_M_′ were recorded at four time points during dark–light–dark transitions: Dark—in the dark just before the light onset; Light—at the end of the 12-h light period; 1–30 min after the light was turned off and 2–5 h in the dark after the 12-h light period. The mean values of points of light, 1 and 2 were normalized and presented as percentages in relation to the dark point (mean values of three measurements ± SE). Different letters represent the significant differences in the mean values according to Tukey HSD post hoc test *p ≤* 0.05.

To investigate a possible relationship between chloroplast movement tracked by the kinetics of T changes (Figure 2 and Figure S3) and photosynthetic efficiency, we determined the diurnal changes in the dynamics of Y(II) and NPQ (Figure 4A). The time course of the light-induced changes in Y(II) showed a much faster response to dark–light–dark transitions compared to T changes described above (Figure 1 and Figure 2). Upon illumination, Y(II) started to decrease from the maximum values in the dark and reached a slightly lower value than in the dark (Figure 4A). At an actinic light intensity of 350 µmol photons m^−2^ s^−1^ Y(II) decreased to about 0.5, accompanied by an NPQ increase (Figure 4A). On the other hand, at the highest light intensity (1400 µmol photons m^−2^ s^−1^) Y(II) decreased drastically together with a strong NPQ increase, probably as a result of zeaxanthin formation. Compared to the T changes (Figure 2), the light-induced decrease in Y(II) was faster and reached the steady-state level within 15 min. While T changes in the first hour upon illumination showed quite variable dynamics strongly dependent on light intensity (Figure 2), the dynamics of Y(II), but not its value, was similar regardless of light intensity. 

Low actinic light had no significant effect on F_0_′ and F_M_′ during the light–dark transition, while at 1400 µmol photons m^−2^ s^−1^, the initial decrease in F_0_′ by about 30% compared to those observed at previous dark period, (Figure 4A), determined immediately after turning light off, was followed by a continuous increase during dark. At medium light, however, the initial increase in F_0_′ by about 40% compared to those observed at the previous dark period (Figure 4A) was followed by its decrease in the subsequent 5 h in dark. In the dark following 12 h at all three light intensities, F_M_′ recovered within 30 min when compared to the values determined immediately before turning the light on (Figure 4C, Table S1).

**Figure 5:**
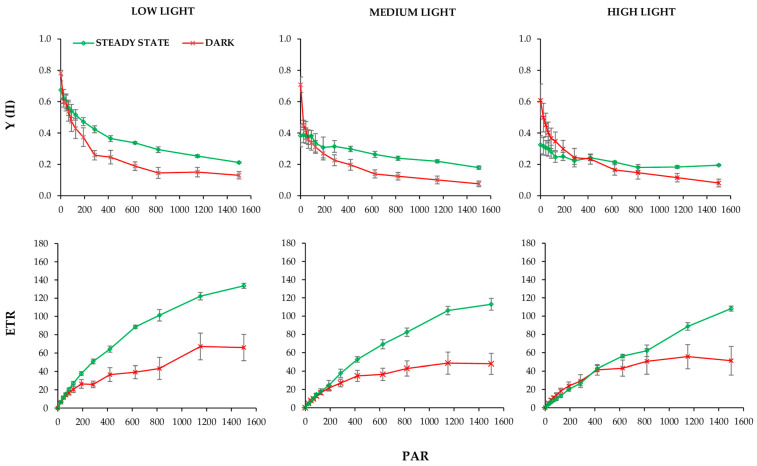
Rapid light curves with replicates (three plants ± SE) at three different time points obtained during the day with *P. zonale* plants grown at three different light intensities: low light, 100 µmol photons m^−2^ s^−1^; medium light, 350 µmol photons m^−2^ s^−1^; high light, 1400 µmol photons m^−2^ s^−1^. PAR, photosynthetically active radiation.

To correlate the effects of photosynthetic induction period as a function of the fully activated Benson–Calvin cycle and to investigate the effects of changes in chloroplast positions on Y(II) and leaf transmittance under red light, we plotted light response curves with very short varied light periods (10 s)—rapid light curve (RLC) (Figure 5). Plants were exposed to increasing light intensities, and the RLC of Y(II) and the calculated electron transport rate (ETR) were recorded at 15 min before the light was switched on, 15 min after the light onset, and at a steady state assuming different chloroplast positions (Figure 5 and Figure S5). In the dark, the chloroplasts are located at the cell bottom. At 15 min after switching on the light, it is assumed that chloroplasts either partially accumulate at low light intensity or reach the peak of avoidance at medium and high light intensities (Figure 2 and Figure S3). In a steady state at different light intensities, RLC showed light-dependent differences, with the most remarkable changes observed at low light. 

### 2.4. Non-Invasive Measurements of Chlorophyll (chl) and Epidermal Flavonoids (Flav)

Medium and high light intensities induced the accumulation of epidermal flavonoids (Flav) in *P. zonale* leaves (Table 1). In parallel, chl content did not change significantly between low and medium light-treated plants, while a significant decrease was observed at high light (Table 1).

### 2.5. Differentially Expressed Genes (DEGs) Involved in Blue-Light Sensing and Chloroplast Movement in GS and WS

Though the observed changes in red-light transmittance were assumed to be mediated by blue-light receptors, phototropins, we performed the experiment using red and blue LED lamps and recorded diurnal changes during several days (Supplementary Figure S6). A characteristic kinetics in T, described above, was observed only when the leaf was illuminated with blue light. Interestingly, the levels of DEGs encoding Phot1 and Phot2 transcripts were higher in WS than in GS (Table 2). A similar result was obtained for DEGs corresponding to the other group of blue light receptors, the cryptochromes, where, out of four DEGs identified, only one was upregulated in GS compared to WS. However, transcripts of DEGs involved in chloroplast movement, such as those encoding chloroplast unusual positioning 1 protein (CHUP1), were differentially expressed in GS and WS (three CHUP1 transcripts were more abundant in WS, and two were more abundant in GS; Table 2.

## 3. Discussion

Our results on the changes in red-light-induced transmittance (T) in the leaves of variegated *P. zonale* plants (Figure 1, Figure 2 and Figures S1–S3) are consistent with the expected light-dependent chloroplast movement previously reported in all plant species, from algae to terrestrial plants [28,41,42]. Under laboratory or field conditions, the chloroplasts’ location in leaf cells of different plant species depends on light intensity [43,44,45]. 

In this study, the variegated leaves of *P. zonale* were used as a good model system to study chloroplast movements in response to different light intensities. To our knowledge, this is the first report on red-light transmission in variegated *P. zonale*, including the green and white leaf sectors (GS and WS). Due to the high sensitivity and good signal-to-noise ratio of the recently developed system for laboratory use, which allows continuous long-term optical measurements in different plant species [36], we were able to detect very small transmittance changes, even oscillations of T in GS, as well as very small T light-induced changes in WS (Figure 1, Figure 2, Figure 3 and Figure S4). Under our experimental conditions, the diurnal T changes under light intensities of 25–350 μmol photons m^−2^ s^−1^ were reversible and repeatable over several days and weeks (Supplementary Figure S1). In contrast, a progressive and irreversible increase in T at light intensities above 1000 μmol photons m^−2^ s^−1^ could indicate possible photoinhibition (Figure 2). 

Comparison of the obtained T changes induced by blue light with those induced by white light in the green and white leaf sectors implied the involvement of phototropins (Figure 2, Figures S3 and S6; Table 2). Thus, the T changes induced by different intensities of white light may be attributed to only 13% contribution of the blue component of the total spectrum of the LED lamps. Although the kinetics of the changes induced by blue light were similar to those induced by about 300 μmol photons m^−2^ s^−1^ of white light, characterised by the first peak preceded by the slow accumulation response, they were much higher and faster than those measured at white light (Supplementary Figure S6).

Despite the absence of developed chloroplasts in the mesophyll layer, the cells in the white leaf sectors showed upregulation of the two blue-light receptors, Phot1 and Phot2. These two receptors are key regulators of chloroplast relocation in photosynthetic leaves [30,31,41]. They translate the light signal into a chemical signal by autophosphorylation, which is controlled by light-oxygen-voltage (LOV)1 and LOV2 domains and leads to the receptor dimerisation [29,31]. Phot1 exhibits greater light sensitivity and higher kinase activity than Phot2 [46]. Polyubiquitination of Phot1 at high intensity of blue light (120 µmol photons m^−2^ s^−1^) triggers its degradation, leading to desensitisation of the receptor [47]. Therefore, the higher abundance of Phot1 and Phot2 transcripts in WS than in GS (Table 2) should be discussed with caution, as the transduction initiated by these receptors depends on the phototropin protein level (defined by post-translational modifications) and dimerisation. 

The physiological significance of the observed T changes in the white leaf sectors is not known. We propose that they are related to chloroplast motility in guard cells adjacent to stomata, which is associated with a phototropin-triggered stomatal opening under blue light [25]. Although plastids do not contain thylakoids or starch granules, numerous membrane vesicles of different sizes and smaller, grouped plastoglobules have been observed in the inner soluble region [37]. However, the epidermis of both leaf tissues of variegated *P. zonale* contains a similar number and size of chloroplasts, particularly in the guard cells [48]. Here, an applied T measurement system gives us the opportunity to apply it in future studies focusing on the mechanisms of stomatal opening under various stresses and during different developmental stages.

At lower light intensities, ˂150 μmol photons m^−2^ s^−1^, the transmittance declined to about 70% of ΔT_DL_ within one hour, followed by a slow decrease throughout the whole light period (Figure 2). This is consistent with the proposed slow chloroplast accumulation reaching steady-state values within ~1.5 h [49,50,51]. The attenuated T oscillations, including a light-induced peak and a drop in T (accumulation response) upon darkening, were observed at the light intensities from 250 to 400 μmol photons m^−2^ s^−1^ (Figure 2 and Figure S3). Our results on the occurrence of the transient avoidance response at this light intensity, follow a similar pattern previously shown in Arabidopsis plants [15]. The biphasic response of Phot1 triggered by higher light intensities and blue light of 16 µmol photons m^−2^ s^−1^, with partial chloroplast avoidance preceding the accumulation phase, has been described previously [52,53]. The kinetics obtained in *P. zonale* leaves can be explained by the fact that both chloroplast accumulation and avoidance occur simultaneously with the avoidance response starting and proceeding faster than the accumulation response, which has a longer lifetime [16,54]. In conclusion, the observed reversible and complex kinetics of T in GS of variegated *P. zonale* indicated that the interplay between accumulation and avoidance response of chloroplasts is required to maintain their optimal position within the cell in terms of photosynthetic efficiency.

In contrast, the highest white light intensity (>1100 µmol photons m^−2^ s^−1^) resulted in a continuous and irreversible T increase, with similar T_L_ and T_D_ values (Figure 2B), without recovery of T_D_, which could indicate loss of chlorophyll and photoinhibition. Decreased chl levels accompanied by the accumulation of epidermal flavonoids (Flav; Table 1), which have a UV-protective role in the leaf [37], confirmed that high light stress occurred under these conditions and was induced by white light without UV-B radiation. We have previously found that high intensity of white light induces differential antioxidative responses in GS and WS, including the distinct stimulation of phenylpropanoid and flavonoid pathways [37,38,39]. The data on the high NPQ observed under the same light regime (Figure 4A) imply that none of the photoprotective mechanisms, including the avoidance response (evidenced by the continuous T increase) was effective in preventing the harmful effects of light excess. This result is consistent with the previously shown induction of antioxidative enzymes and accumulation of H_2_O_2_ in the apoplast of vascular and (peri)vascular tissues only in GS of the *P. zonale* leaf under high intensity of white light (>1100 μmol photons m^−2^ s^−1^) [38].

These results argue against the widely accepted essential role of the avoidance response in the escaping photodamage [16,17,18]. Alternatively, the avoidance of high light intensity by translocation of chloroplasts along the anticlinal cell wall has been explained by better light penetration into the otherwise shaded deeper mesophyll layers, which could be important for species with tick leaf [19,55,56,57,58].

To investigate the role of chloroplast distribution patterns in leaf photosynthesis, we correlated Y(II) and NPQ changes with observed light-dependent T kinetics (Figure 1, Figure 3 and Figure 4). Based on the T kinetics and Y(II) measured simultaneously at the same leaf spot, we could not confirm the relationship between Y(II) and chloroplast positioning based on determined T and Y(II) kinetics. The Y(II) changes were always faster and preceded the slow T changes. At low light, leaves showed reduced NPQ activity, whereas at high light, high NPQ accompanied by low ETR, and lower F_M_′ and F_0_′, implied possible photoinhibition (Figure 4B). In a comprehensive study, Pfündel and authors [32] investigated in detail the effects of blue and red light, as well as their combination on the chlorophyll fluorescence parameters (F_0_′, F_M_′), and suggested that besides absorption variations, there is another specific effect of blue light, which moderates excitation pressure at the PSII. This result does not support the proposal that chloroplast accumulation helps maximise light harvesting under energy-limiting conditions, either by altering light absorbance or excitation pressure in the PSII as suggested elsewhere [45,59,60]. However, the analysis of the rapid light curves at different time points at the same leaf spot combined with the obtained T changes caused by: (i) chloroplasts distribution in dark; (ii) chloroplast accumulation 15 min upon illumination; and (iii) chloroplast accumulation 2 h after illumination (Figure 1, Figure 2, Figure 3, Figures S3 and S4), showed that initial Y(II) and ETRmax responded to the chloroplasts’ accumulation response as expected [35]. Therefore, the lowest initial slope of ETR measured in the dark-adapted chloroplast position of the plant exposed to low light intensity implied that the limitation of light harvesting and excitation pressure in the PSII was caused by shading of chloroplasts within a cell.

However, the rapid light curves obtained from the same leaf area upon illumination showed a gradual decrease in Y(II) with increasing irradiance and a gradual recovery of both parameters and ETRmax. As a consequence, photosynthetic capacity increased significantly, which may be partly influenced by the movement of chloroplasts to the periclinal position (Figure 4). With increasing light intensity: 300 and 1400 μmol photons m^−2^ s^−1^, the difference between the rapid light curves measured at the same time points decreased compared to plants grown at low light, but this was accompanied by lower ETR values at higher saturating light intensities. At 1400 μmol photons m^−2^ s^−1^, the lowest ETR was determined at saturated light intensities, indicating either an avoidance response or even photoinhibition. 

## 4. Materials and Methods

### 4.1. Plant Material and Growth Conditions 

The model plant used in this experiment was the variegated *P. zonale*, cultivar “Frank Headley” [61]. Ten weeks before the start of the experiment, the cuttings were vegetatively propagated by cuttings from the “mother plants” in small pots (6 × 6 × 5 cm) with Substrate 2 (Klasmann-Decilmann, Geeste, Germany) in the greenhouse. After one month, the plantlets were transferred to the growth chamber at 25/22 °C day/night temperature with a 16-h photoperiod and 180 µmol photons m^−2^ s^−1^ white light for molecular studies. For the transmittance and chl fluorescence measurements, the plants were transferred to the growth chamber, the interior of which is coated with mylar foil that reflects up to 98% of the light. Plants were grown on a 12 h photoperiod at 23/20 °C day/night temperature and relative humidity of 60 ± 5%. We used three LED dimmable panels with continuous illumination (Samsung LED LM301H Quantum Tech V3 Panel Light 240W, Yeongtong-gu, Suwon, South Korea). The panels were mounted in a temperature-controlled box with a specially designed fan system to control the heating of the panel and continuously change the light intensity according to the experimental requirements. The plants were exposed to five different white light intensities: (I) 25 ± 4 µmol photons m^−2^ s^−1^; (II) 140 ± 17 µmol photons m^−2^ s^−1^; (III) 290 ± 40 µmol photons m^−2^ s^−1^; (IV) 290 ± 40 μmol photons m^−2^ s^−1^; (V) 1200 ± 80 µmol photons m^−2^ s^−1^. The spectrum of LED panels is shown in Supplementary Figure S7.

### 4.2. Transmittance Measurements

Recently, we have described a non-destructive measurement system that can track the optical transmission of leaves in real-time dependence [36]. Briefly, the experimental apparatus has 20 independent “channels”, each channel corresponding to a single leaf. The light source for each channel is a red signal LED with a spectral emission maximum at 656 nm. Each signal LED was connected to an optical fibre. The other end of this fibre was attached to the specific leaf holder made of transparent plastic (methyl methacrylate). The two additional optical fibres, one collecting transmitted and one collecting reflected light, were also integrated and attached to a leaf holder. The optical fibres transmit the collected light to the photodiodes, which are placed in a thermostatic shielded box. The signals from the photodiodes were routed to the precision digital multimeter (DMM) (HP 34970A, Agilent Technologies, Santa Clara, CA, USA). The DMM was connected to the computer via the interface RS-232. The leaf holder was designed to provide five degrees of freedom for mechanical movement, allowing changes in the leaf’s position and orientation [36]. The channels were distributed into six independent groups, each group containing three individual channels, while the remaining two channels were used to control the overall system dynamics with neutral filters. Each plant group was exposed to different light regimes. Calibration is presented in Supplementary Figure S8. 

### 4.3. Chlorophyll Fluorescence Parameters 

The efficiency of PSII was calculated according to measurements of the modulate pulse chlorophyll fluorescence using the portable chlorophyll fluorimeter Junior PAM (Gadermann Instruments GmbH, Würzburg, Germany). Photochemical activity was calculated using WinControl software (v3.29; Heinz Walz GmbH, Effeltrich, Germany) and the photochemical quantum efficiency of PSII as described by Van Kooten and Snel [62]. The measurements were performed continuously during 24 h by applying a saturating pulse (10,000 μmol photons m^−2^ s^−1^) every 20 min. The minimum fluorescence (F_0_) and the maximum fluorescence (F_M_) were measured in dark-adapted leaves, and the maximum photochemical quantum yield of PS II, Y(II), was calculated as F_v_/F_M_; (F_v_ = F_M_ − F_0_) (Junior PAM Manual, p. 96). The maximal chl fluorescence (F_M_′) and chl fluorescence (F′) in light-adapted leaves and the effective photochemical quantum yield of PSII, Y(II) = (F_M_′ − F′)/F_M_′, was estimated as according to Genty et al. [63]. However, it should be noted that the correctness of the model has been questioned recently [64]. Non-photochemical quenching (NPQ) was calculated according to the Stern–Volmer equation NPQ = (F_M_ – F_M_′)/F_M_′ [65]. Rapid light curves provide information about the current photosynthesis state and should not be influenced by photosynthetic rates at steady state, as the light duration of each actinic light was 10 s. The fluorescence parameters of RLCs taken from the individual plant present mean values of three leaves, acclimatised to three white light intensities (photosynthetically active radiation, PAR): low light (LL, 100 µmol photons m^−2^ s^−1^); medium light (ML, 350 µmol photons m^−2^ s^−1^); and high light intensity (HL, 1400 µmol photons m^−2^ s^−1^). The relative electron transport rate (ETR) is calculated as follows: ETR = PAR ETR_Factor, 0.84 × PPS2/PPS1 × Y(II). The RLC provides the following parameters: (i) α–initial slope of RLC, which is related to quantum efficiency of photosynthesis; (ii) ETRmax—maximum electron transport rate, expressed in µmol photons electrons m^−2^ s^−1^.

The photochemical activity was measured at the same leaf spots used for the T measurements and varied by 20% in the light, thus enabling that the T measurements with the red-light beam had no significant effect on the chl excitation in the modulated chl fluorescence measurements in the light.

### 4.4. Dertermination of chl, Flav and NBI 

Total chlorophyll concentration (chl), epidermal flavonoids (Flav), and the leaf nitrogen balance index (NBI) were determined using the Dualex sensor (Dx4, FORCE-A, Orsay, France; [66]). Two laser beams, at 375 and 650 nm, were directed at the leaf surface using a leaf clip, to excite the chl in the leaf mesophyll. By equalising the chl fluorescence under visible (650 nm) and UV (375 nm) light and electronic feedback loop, variable chl fluorescence is avoided, and an accurate measurement of the absorbance of Flav in the UV range is secured. The NBI is derived from the Chl/Flav ratio and is an indicator for the carbon/nitrogen ratio. Measurements were obtained from the same leaf spot used for the chl fluorescence and T measurements.

### 4.5. Annotation of the Subcellular Localisation of Photoreceptors in P. zonale 

Very recently, we performed a de novo transcriptome analysis of *P. zonale* and, for the first time, analysed the differentially expressed genes (DEGs) in GS and WS. Prediction of the subcellular location of a protein encoded by selected DEGs (published in [54]) was conducted by WoLF-PSORT tool [66].

### 4.6. Statistics

The statistical significance of the content of chl and epidermal flavonoids, as well as NBI of *P. zonale* GS under different light intensities, was evaluated by one-way ANOVA. Homogeneity of variance was tested using Levene’s test, and Tukey’s post hoc test was used to test for significant differences in chl and epidermal flavonoids and NBI between the different treatment groups. The threshold for significance was set at 0.05.

The statistical significance of normalised F_0_′ and F_M_′ of *P. zonale* GS under different light intensities and time points was evaluated by two-way ANOVA. Homogeneity of variance was tested using Levene’s test and original values, and Tukey’s post hoc test was used to test for significant differences in normalised F_0_′ and F_M_′ of *P. zonale* GS measured at different PAR and time points. The threshold for significance was set at 0.05.

## 5. Conclusions

The reversible and complex kinetics of red-light transmittance in green and white leaf sectors of variegated *P. zonale* indicate the interplay between chloroplast accumulation and avoidance response, which strongly depends on the intensity of white light. Similar kinetics were obtained with blue-light-induced transmittance, implying the involvement of phototropins. The observed differential expression of phototropins, cryptochromes and CHUP1 between white and green leaf sectors requires further investigation at protein and post-translational levels to identify their involvement in chloroplast movement. Our analysis of chlorophyll fluorescence parameters simultaneously with transmittance changes indicates that chloroplast position influences the quantum efficiency of PSII photochemistry. However, a strong avoidance response, together with other photoprotective mechanisms under high light intensity, was not efficient in maintaining the high rate of the photosynthetic electron transport.

## Figures and Tables

**Table 1:** Concentrations of total chlorophyll (Chl), epidermal flavonoids (Flav) and leaf nitrogen balance index (NBI) in the GS adaxial epidermis of *P. zonale* plants at the end of experiment presented in Figure 4.

White Light Regime	Chl (µg cm^−2^)	Flav, (µg cm^−2^)	NBI
Low light—LL	20.62 ± 0.74 ^a^	0.16 ± 0.01 ^a^	127.72 ± 0.88 ^c^
Medium light—ML	21.54 ± 2.68 ^a^	0.54 ± 0.01 ^b^	47.36 ± 3.52 ^b^
High light—HL	14.94 ± 1.02 ^a^	0.92 ± 0.09 ^c^	17.11 ± 3.11 ^a^

Results represent the mean value from 4–6 leaves per plant, taken from three plants ± SE. Different letters indicate statistically significant differences between different light intensities. Low light, 100 µmol photons m^−2^ s^−1^; medium light, 350 µmol photons m^−2^ s^−1^; high light, 1400 µmol photons m^−2^ s^−1^.

**Table 2:** Differentially expressed genes (DEGs) related to blue-light receptors and chloroplast unusual positioning 1 (CHUP1).

Annotated Transcript	GS Value	WS Value	log2FC	*p* Value	*p* Adjust	Compartment
phototropin 1 (Phot1)	0.554948	24.48923	−5.46	2.23 × 10^−5^	0.000759	nucleus
phototropin 2 (Phot2)	5.424345	36.23852	−2.74	1.58 × 10^−9^	2.62 × 10^−7^	plastid
cryptochrome DASH	0.927768	6.097461	−2.72	9.55 × 10^−9^	1.28 × 10^−6^	plastid
cryptochrome DASH	0.016507	0.18674	−3.49	9.69 × 10^−4^	1.37 × 10^−2^	plastid
cryptochrome-1 isoform X1	15.17865	5.070029	1.58	8.40 × 10^−5^	2.13 × 10^−3^	cytosol
cryptochrome-1 isoform X2	0.036141	0.285204	−2.97	2.29 × 10^−3^	0.025591	nucleus
CHUP1	0.014838	0.378728	−4.68	2.74 × 10^−6^	0.000143	plastid
	0.676587	6.034586	−3.16	1.52 × 10^−6^	8.91 × 10^−5^	plastid
	7.423503	30.91456	−2.06	0.000109	0.002619	plastid
	0.327659	0.01742	4.23	0.002075	0.023855	plastid
	0.982411	0.023832	5.37	1.92 × 10^−5^	0.000675	plastid

Data were obtained with three biological replicates of GS and WS (see Materials and Methods section, Section 4).

## Data Availability

The data presented in this study are available in Supplementary Materials.

## Supplementary Materials

The following supporting information can be downloaded at: https://www.mdpi.com/article/10.3390/ijms241814265/s1.
