# Supplementary material for: Red-Light Transmittance Changes in Variegated Pelargonium zonale—Diurnal Variation in Chloroplast Movement and Photosystem II Efficiency
Source: Int J Mol Sci. 2023 Sep 19;24(18):14265. doi: 10.3390/ijms241814265 (PMC10532150; doi:10.3390/ijms241814265)
Supplement: Supplementary file 1 [file ijms-24-14265-s001.zip › Figure S2.pdf]

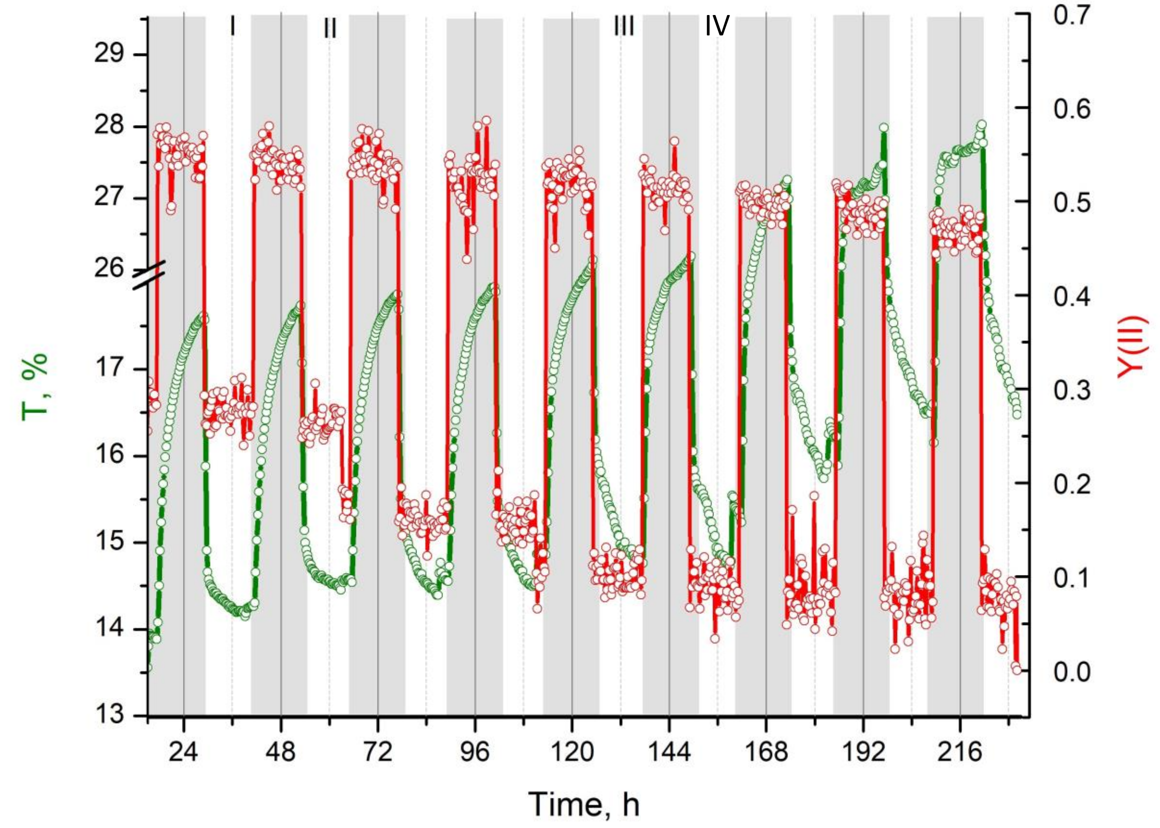

Figure S2. Changes in red light transmittance of (665 nm) induced by white light of different intensities (I–IV). A percentage of total T and Y(II) were determined at the same leaf spot during light-dark-light transitions according to the circadian rhythm. The intensity of white light was I: ~25 µmol m^{-2} s^{-1}; II: ~140 µmol m^{-2} s^{-1}: III: ~290 µmol m^{-2} s^{-1} and IV: ~350 µmol m^{-2} s^{-1} at the leaf level.
