# Supplementary material for: Red-Light Transmittance Changes in Variegated Pelargonium zonale—Diurnal Variation in Chloroplast Movement and Photosystem II Efficiency
Source: Int J Mol Sci. 2023 Sep 19;24(18):14265. doi: 10.3390/ijms241814265 (PMC10532150; doi:10.3390/ijms241814265)
Supplement: Supplementary file 1 [file ijms-24-14265-s001.zip › Figure S3.pdf]

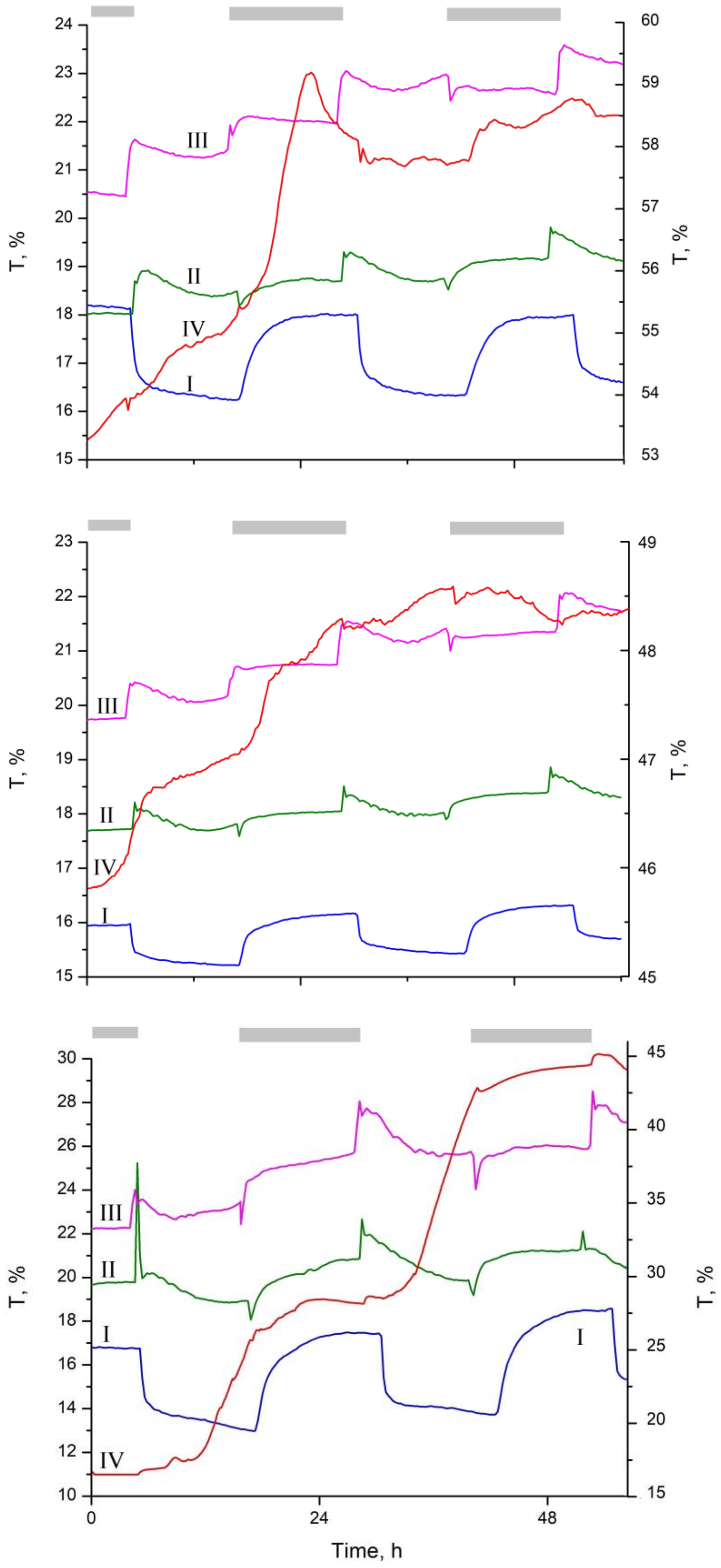

Figure S3. Red light-induced changes in three different biological plants were recorded for different light intensities (I: ~25 µmol m^{-2} s^{-1}; II: ~140 µmol m^{-2} s^{-1}: III: ~290 µmol m^{-2} s^{-1} and IV: ~350 µmol m^{-2} s^{-1} at the leaf level). Transmittance was recorded every 15 minutes. Light intensity IV is shown on the secondary axis. The results of three different plants are presented.
