# Supplementary material for: Red-Light Transmittance Changes in Variegated Pelargonium zonale—Diurnal Variation in Chloroplast Movement and Photosystem II Efficiency
Source: Int J Mol Sci. 2023 Sep 19;24(18):14265. doi: 10.3390/ijms241814265 (PMC10532150; doi:10.3390/ijms241814265)
Supplement: Supplementary file 1 [file ijms-24-14265-s001.zip › Figure S4.pdf]

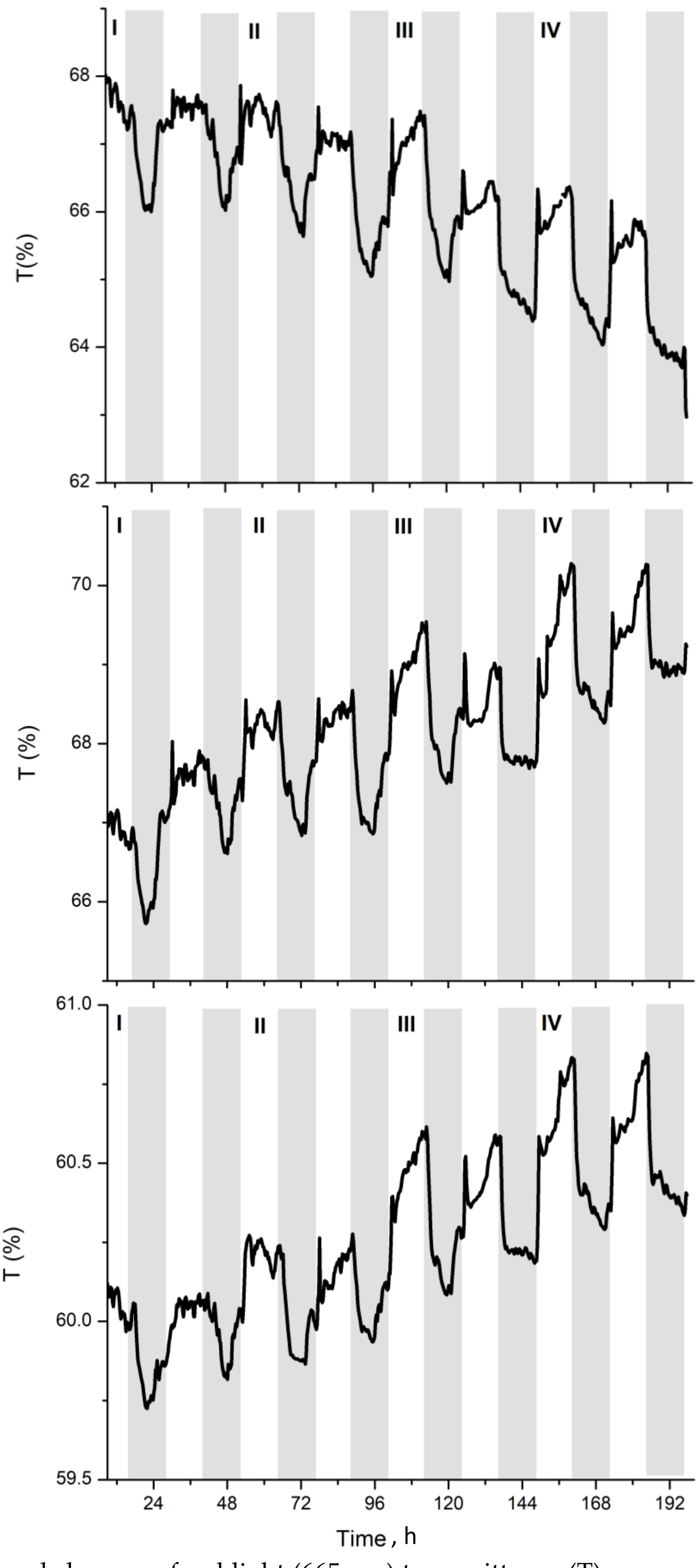

Figure S4. Diurnal changes of red light (665 nm) transmittance (T) expressed as a percentage of the total transmittance of the white sectors of *P. zonale* leaves. Three replicates (from three different plants) of the curve shown in Figure 3 are presented. The transmittance was recorded every 15 min. During the two-day measurement of T, *P. zonale* plants were grown at five light intensities: I: 25 μmol m^{-2} s^{-1}; II: 140 μmol m^{-2} s^{-1}; III: 290 μmol m^{-2} s^{-1}; IV: 350 μmol m^{-2} s^{-1}. Dark periods (12 h) are shown in grey and light periods in white.
