# Supplementary material for: Red-Light Transmittance Changes in Variegated Pelargonium zonale—Diurnal Variation in Chloroplast Movement and Photosystem II Efficiency
Source: Int J Mol Sci. 2023 Sep 19;24(18):14265. doi: 10.3390/ijms241814265 (PMC10532150; doi:10.3390/ijms241814265)
Supplement: Supplementary file 1 [file ijms-24-14265-s001.zip › Figure S5.pdf]

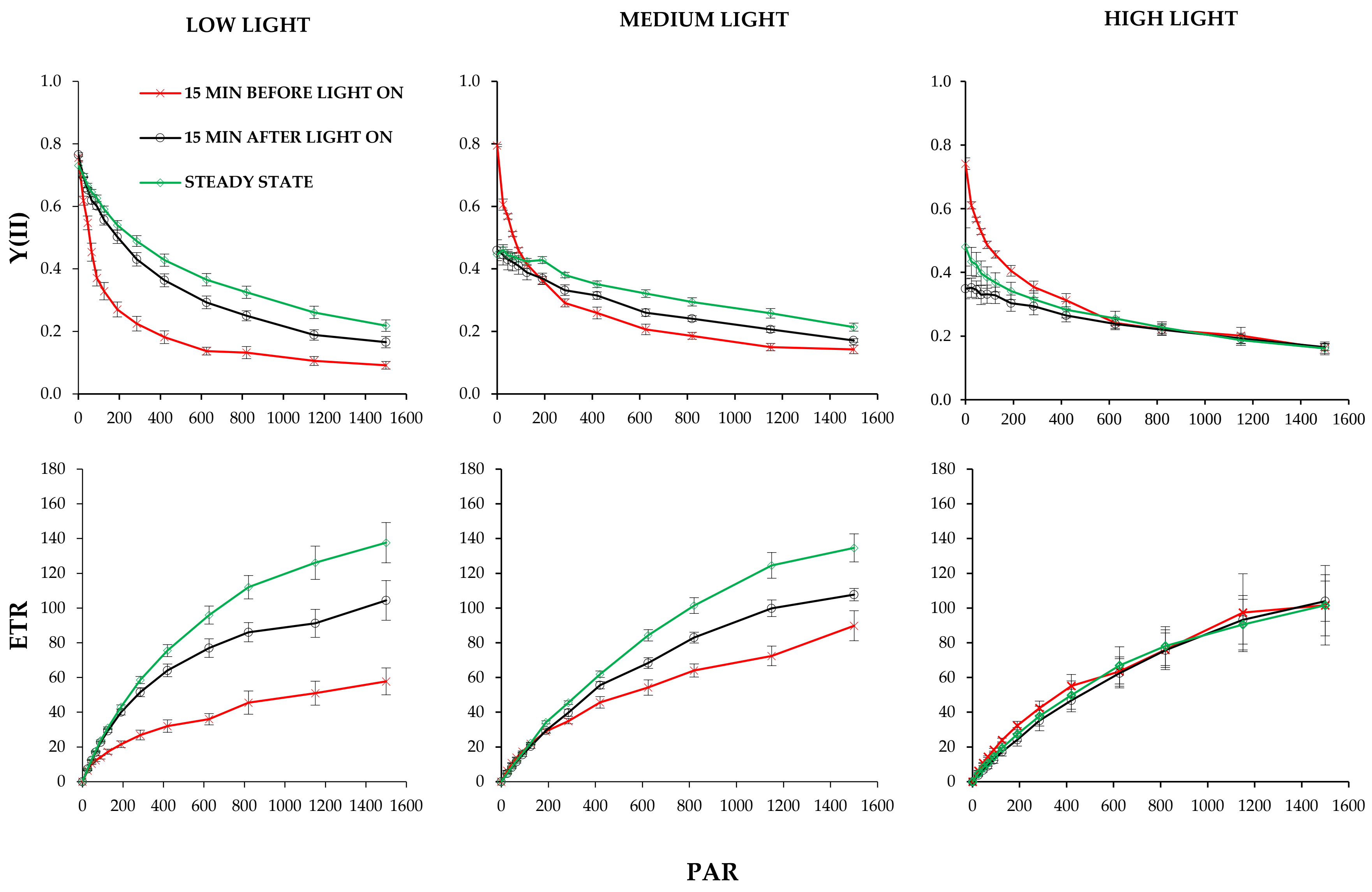

Figure S5. Rapid light curves at three different time points obtained during the day with *P. zonale* plants grown at three different light intensities: low light, 100 µmol $m^{-2} s^{-1}$; medium light, 350 µmol $m^{-2} s^{-1}$; high light, 1400 µmol $m^{-2} s^{-1}$. Results represent the mean of 3 leaves ± SE. PAR, photosynthetically active radiation; Y(II) maximum efficiency of the photosystem II; ETR, electron transport rate.
