# Supplementary material for: Red-Light Transmittance Changes in Variegated Pelargonium zonale—Diurnal Variation in Chloroplast Movement and Photosystem II Efficiency
Source: Int J Mol Sci. 2023 Sep 19;24(18):14265. doi: 10.3390/ijms241814265 (PMC10532150; doi:10.3390/ijms241814265)
Supplement: Supplementary file 1 [file ijms-24-14265-s001.zip › Figure S6.pdf]

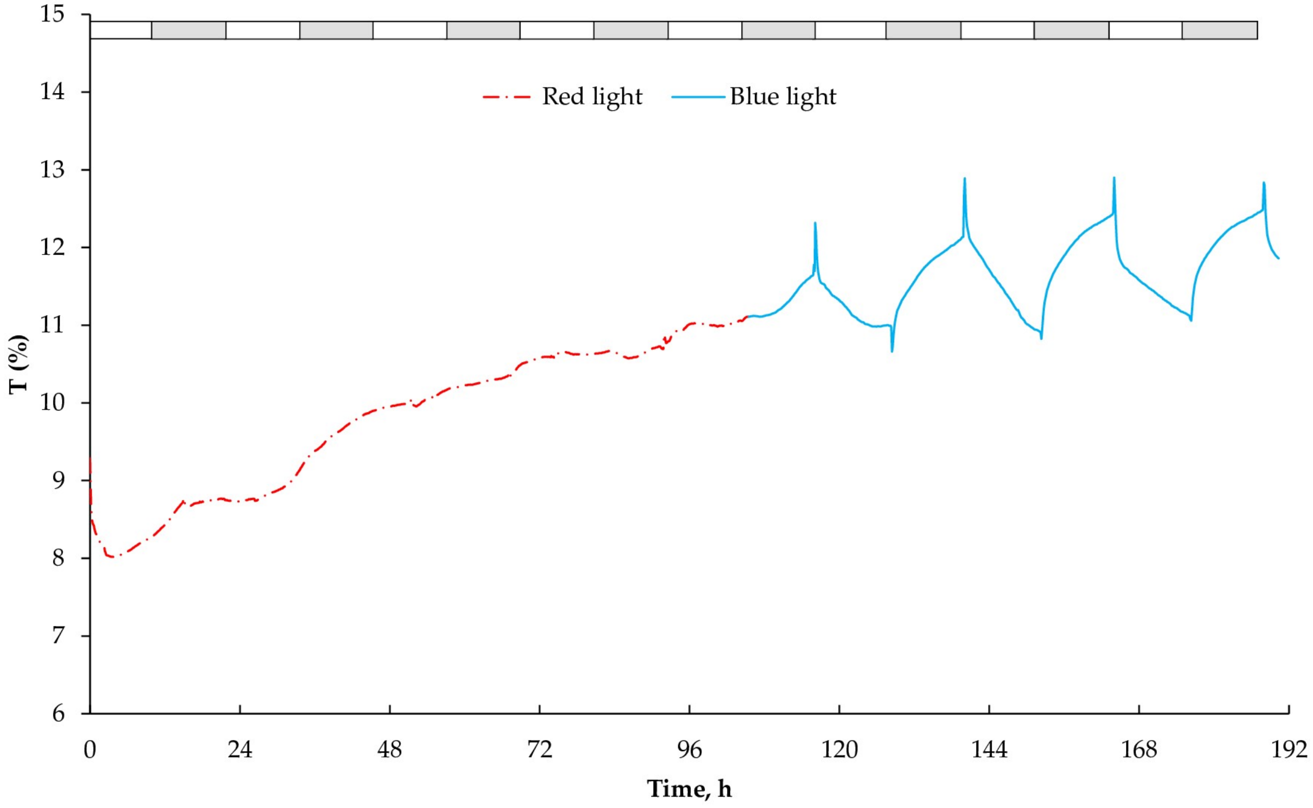

Figure S6. Changes in red light transmittance in green leaf sectors of *P. zonale* plants induced by red (656 ± 16 nm) and blue (443 ± 30 nm) LED lamps both at 400 µmol m^{-2} s^{-1}.
