# Supplementary material for: Red-Light Transmittance Changes in Variegated Pelargonium zonale—Diurnal Variation in Chloroplast Movement and Photosystem II Efficiency
Source: Int J Mol Sci. 2023 Sep 19;24(18):14265. doi: 10.3390/ijms241814265 (PMC10532150; doi:10.3390/ijms241814265)
Supplement: Supplementary file 1 [file ijms-24-14265-s001.zip › Figure S7.pdf]

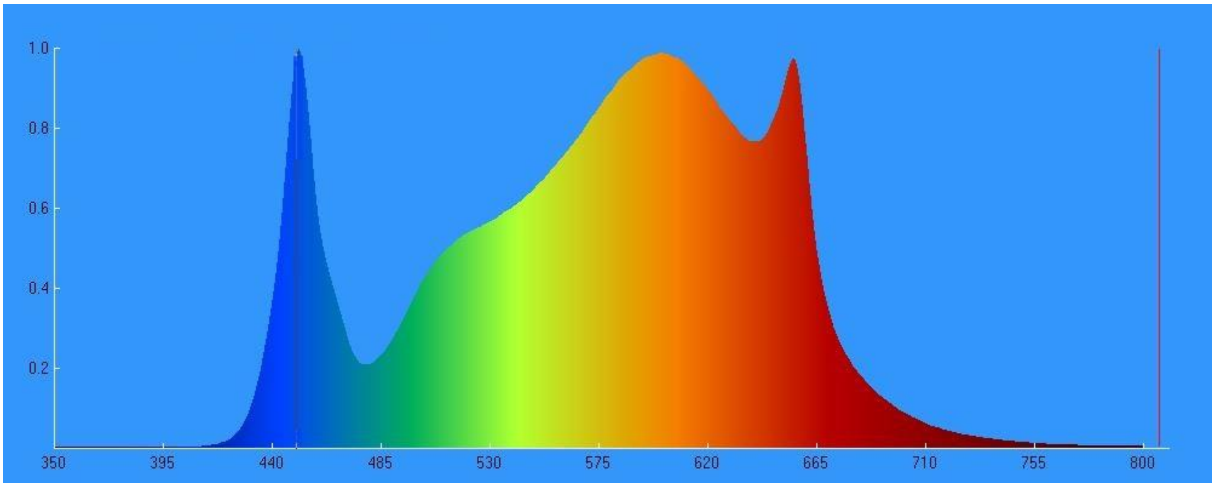

Figure S7. The spectrum of actinic light is provided by LED dimmable panels with continuous illumination (model Samsung LED LM301H Quantum Tech V3 Panel Light 240W, Yeongtong-gu, Suwon, South Korea).
