# Supplementary material for: Red-Light Transmittance Changes in Variegated Pelargonium zonale—Diurnal Variation in Chloroplast Movement and Photosystem II Efficiency
Source: Int J Mol Sci. 2023 Sep 19;24(18):14265. doi: 10.3390/ijms241814265 (PMC10532150; doi:10.3390/ijms241814265)
Supplement: Supplementary file 1 [file ijms-24-14265-s001.zip › Figure S8.pdf]

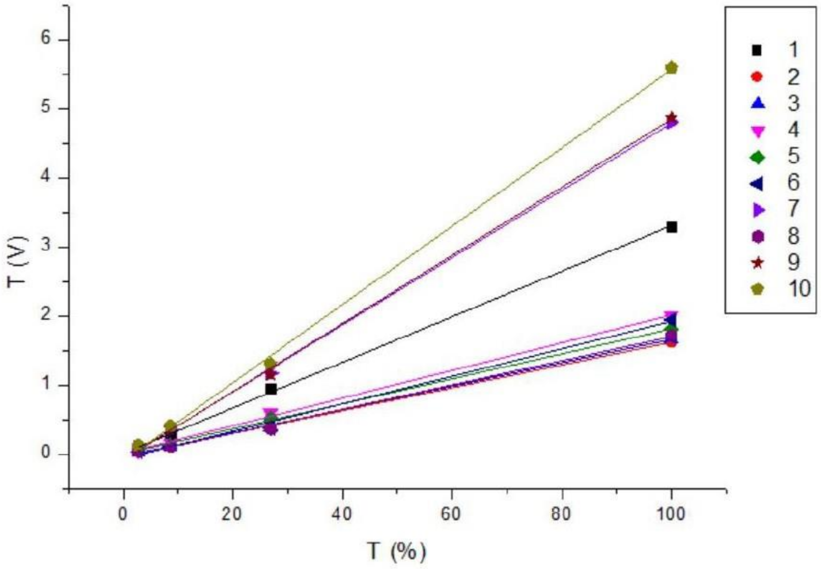

Equation	y = a + b*x		
Weight	No Weighting		
Residual Sum of Squares	0.00409	0.00168	0.0012
Pearson's r	0.99969	0.99949	0.99965
Adj. R-Square	0.99906	0.99846	0.99895
		Value	Standard Error
T	Intercept	0.01425	0.03026
T	Slope	0.03292	5.82335E-4
T	Intercept	-0.02613	0.01938
T	Slope	0.01648	3.72939E-4
T	Intercept	-0.02723	0.01642
T	Slope	0.0169	3.15937E-4
T	Intercept	0.0137	0.02607
T	Slope	0.01998	5.01707E-4
T	Intercept	0.00827	0.01698
T	Slope	0.01805	3.26717E-4
T	Intercept	-0.06509	0.04796
T	Slope	0.01989	9.23026E-4
T	Intercept	-0.06743	0.03785
T	Slope	0.04854	7.28414E-4
T	Intercept	-0.04498	0.03188
T	Slope	0.01747	6.1365E-4
T	Intercept	-0.07385	0.05358
T	Slope	0.0492	0.00103
T	Intercept	-0.0925	0.06708

Figure S8. The graph shows calibration curves consisting of 10 linear lines, representing the measured values obtained with a set of calibrated neutral density filters (NDFs). Each measurement location has unique parameters "a" and "b" that are different for each individual channel. These parameters were used to calculate the light transmission in percentages (%). By measuring the transmitted signals for each filter in the NDF set for each individual channel, the relative sensitivities of the channels were determined. Subsequently, these measured sensitivities were employed as correction factors, to align the measured values of each channel on a uniform (relative) scale.
