# Supplementary material for: Red-Light Transmittance Changes in Variegated Pelargonium zonale—Diurnal Variation in Chloroplast Movement and Photosystem II Efficiency
Source: Int J Mol Sci. 2023 Sep 19;24(18):14265. doi: 10.3390/ijms241814265 (PMC10532150; doi:10.3390/ijms241814265)
Supplement: Supplementary file 1 [file ijms-24-14265-s001.zip › Table S1.pdf]

Table S1. Two-way ANOVA results for the effects of time point (as described in Figure 4B and 4C) and PAR (LL, ML and HL) as well as their interaction on the relative values of normalised F_0' and F_M' measured on the leaves of *P. zonale* plants. The *Df*s are shown in the brackets (the first number represents *Df* of main effects and their interactions and the second number is *Df* of error). F_0' and F_M', chlorophyll fluorescence parameters.

Trait	Source of variation	*F*	*P* > *F*	Trait	Source of variation	*F*	*P* > *F*
**F_0'**	**Time point**	**3.21**	**0.017**	**F_M'**	**Time point**	**18.86**	**< 0.0001**
(*Df*: 1; 36)	**PAR**	**38.30**	**< 0.0001**	(*Df*: 1; 24)	**PAR**	**3.97**	**0.032**
	**Time point × PAR**	**3.51**	**0.003**		**Time point × PAR**	**6.82**	**< 0.0001**

ANOVA - Fo

Cases	Sum of Squares	df	Mean Square	F	p
Time point	2660.424	5	532.085	3.212	0.017
Light intensity	12691.158	2	6345.579	38.302	< .001
Time point ✻ Light intensity	5813.651	10	581.365	3.509	0.003
Residuals	5964.260	36	165.674		

Note. Type I Sum of Squares

Cell No.	Time Point	PAR	Fo Mean	1	2	3
8	Point_2	ML	75.4667	****		
5	Point_1	ML	75.6667	****		
11	Point_3	ML	75.7333	****		
14	Point_4	ML	75.9667	****		
17	Point_5	ML	80.8000	****	****	
13	Point_4	LL	88.0667	****	****	
10	Point_3	LL	88.7333	****	****	
7	Point_2	LL	89.5000	****	****	
4	Point_1	LL	91.7333	****	****	
16	Point_5	LL	92.4667	****	****	
2	Light	ML	100.0000	****	****	
3	Light	HL	100.0000	****	****	
1	Light	LL	100.0000	****	****	
6	Point_1	HL	105.1667	****	****	
9	Point_2	HL	109.1667	****	****	
12	Point_3	HL	113.0000	****	****	
15	Point_4	HL	117.2333		****	
18	Point_5	HL	158.8333			****

ANOVA - Fm

Cases	Sum of Squares	df	Mean Square	F	p
Time point	6498.020	3	2166.007	18.864	1.669×10^{-6}
Light intensity	910.842	2	455.421	3.966	0.032
Time point ✻ Light intensity	4697.007	6	782.834	6.818	2.583×10^{-4}
Residuals	2755.747	24	114.823		

Note. Type III Sum of Squares

Cell No.	Time Point	PAR	Fm Mean	1	2	3
6	Light	HL	37.2667			****
5	Light	ML	77.1667	****		
9	Point_1	HL	85.5333	****	****	
10	Point_2	LL	90.6000	****	****	
4	Light	LL	90.6667	****	****	
7	Point_1	LL	95.3333	****	****	
8	Point_1	ML	98.2000	****	****	
2	Dark	ML	100.0000	****	****	
1	Dark	LL	100.0000	****	****	
3	Dark	HL	100.0000	****	****	
11	Point_2	ML	103.5667	****	****	
12	Point_2	HL	112.3333		****	
